# Improving Delivery Accuracy of Stereotactic Body Radiotherapy to a Moving Tumor Using Simplified Volumetric Modulated Arc Therapy

**DOI:** 10.1371/journal.pone.0158053

**Published:** 2016-06-22

**Authors:** Young Eun Ko, Byungchul Cho, Su Ssan Kim, Si Yeol Song, Eun Kyung Choi, Seung Do Ahn, Byongyong Yi

**Affiliations:** 1 Department of Radiation Oncology, Ulsan University Hospital, Ulsan, Korea; 2 Department of Radiation Oncology, Asan Medical Center, University of Ulsan College of Medicine, Seoul, Korea; 3 Department of Radiation Oncology, University of Maryland School of Medicine, Baltimore, Maryland, United States of America; University of California, San Diego, UNITED STATES

## Abstract

**Purpose:**

To develop a simplified volumetric modulated arc therapy (VMAT) technique for more accurate dose delivery in thoracic stereotactic body radiation therapy (SBRT).

**Methods and Materials:**

For each of the 22 lung SBRT cases treated with respiratory-gated VMAT, a dose rate modulated arc therapy (DrMAT) plan was retrospectively generated. A dynamic conformal arc therapy plan with 33 adjoining coplanar arcs was designed and their beam weights were optimized by an inverse planning process. All sub-arc beams were converted into a series of control points with varying MLC segment and dose rates and merged into an arc beam for a DrMAT plan. The plan quality of original VMAT and DrMAT was compared in terms of target coverage, compactness of dose distribution, and dose sparing of organs at risk. To assess the delivery accuracy, the VMAT and DrMAT plans were delivered to a motion phantom programmed with the corresponding patients’ respiratory signal; results were compared using film dosimetry with gamma analysis.

**Results:**

The plan quality of DrMAT was equivalent to that of VMAT in terms of target coverage, dose compactness, and dose sparing for the normal lung. In dose sparing for other critical organs, DrMAT was less effective than VMAT for the spinal cord, heart, and esophagus while being well within the limits specified by the Radiation Therapy Oncology Group. Delivery accuracy of DrMAT to a moving target was similar to that of VMAT using a gamma criterion of 2%/2mm but was significantly better using a 2%/1mm criterion, implying the superiority of DrMAT over VMAT in SBRT for thoracic/abdominal tumors with respiratory movement.

**Conclusion:**

We developed a DrMAT technique for SBRT that produces plans of a quality similar to that achieved with VMAT but with better delivery accuracy. This technique is well-suited for small tumors with motion uncertainty.

## Introduction

Stereotactic body radiation therapy (SBRT) delivering an ablative high dose to tumors is highly effective for controlling individual metastases or early-stage, non-metastatic primary tumors [1–4]. In order to deliver a large fractional dose to the tumor while minimizing normal tissue toxicity, both highly conformal dose delivery at the tumor site and rapid dose fall-off away from the target are key requirements for SBRT.

A wide spectrum of SBRT techniques that use modern treatment machines in which the image guidance capability enables high precision tumor targeting is available, from three-dimensional conformal radiotherapy (3DCRT) to intensity modulated radiotherapy (IMRT) or volumetric modulated arc therapy (VMAT) [5].

In 3DCRT, 7–11 static non-coplanar beams are used to achieve highly conformal dose distribution. However, it requires more time to plan the customized beam arrangement and deliver the treatment because of the variability in gantry, collimator, and couch positions.

Tumor motion due to respiration is an additional challenge in SBRT for thoracic and abdominal tumors. For free-breathing delivery, the irradiation volume should be large enough not to miss the tumor at any point of the respiratory cycle. Further, a necessary margin to account for setup uncertainties can lead to an irradiation volume that is 2–3 times larger than the tumor volume itself. Several strategies such as active breath holding [6], abdominal compression [7], and respiratory gating [8] have been employed to counter the effect of respiratory motion. However, these may not be sufficient to obtain high conformal dose delivery at the tumor site as well as a rapid fall-off of the dose to the surrounding healthy tissues.

In pursuit of the maximal dose differential between the tumor and surrounding normal tissue, techniques comprising intensity modulation and the shortening of treatment time using coplanar arc delivery have been developed. The more technologically advanced IMRT or VMAT is especially used for thoracic/abdominal SBRT. IMRT and VMAT usually confer a dosimetric advantage over 3DCRT, especially when the target volume is geographically complex or is in close proximity to vital structures [9]. However, the more technically demanding IMRT and VMAT increase treatment complexity by involving the dynamic control of multileaf collimator (MLC) leaf positions. There is a concern that the dynamic complexity of the treatment techniques could increase the uncertainty of dose delivery. For lung or abdominal tumors particularly, their respiratory-induced movement can interplay with the dynamic MLC leaf motion of IMRT and VMAT and result in significant hot and cold spots in the tumor. Although the interplay effect might be clinically ignorable [10], some deviations from the planned dose are inevitable. Moreover, its unpredictable characteristics may affect the safe use of SBRT in cases where a large fractional dose is required.

Unlike IMRT/VMAT, in which a small portion of the tumor is sequentially irradiated, 3DCRT irradiates the whole tumor volume at once with their open fields of uniform intensity. Therefore, it precludes any interplay between the MLC leaf motion and tumor respiratory motion, is less affected by patient movement during a treatment session, and is less amenable to MLC positioning errors. 3DCRT, as a result, is expected to minimize any discrepancy between the planned and delivered treatment, especially when there is large respiratory motion or significant modulation in IMRT and VMAT.

As aptly described in a recent debate [11] on the appropriate technique for lung SBRT, “a good SBRT technique not only generates good plans but also delivers them without deviations.” In this regard, we aimed to develop an SBRT technique that has a plan quality comparable to that of IMRT and VMAT, and that is as simple to implement as 3DCRT to improve accuracy of dose delivery.

In this study, we present a novel SBRT technique that adds the dose rate modulation capability to dynamic conformal arc beam delivery or, equivalently, that eliminates the intensity modulation of MLC from VMAT. In such a way, the technique can deliver a highly conformal dose to the tumor while minimizing the dose to the normal tissues without any concern regarding the interplay effect. The performance of the proposed technique is demonstrated from both plan quality and delivery accuracy perspectives.

## Materials and Methods

### 1. Patients selection and four-dimensional computed tomography (4DCT) simulation

A total of 22 lung cancer patients treated using VMAT SBRT from Jun 2013 to January 2014 [12] were selected for the study. After taking into account the location and size of the tumor and its range of respiratory motion, 10 VMAT cases with respiratory gating and 12 cases without gating were enrolled. This study was approved by the Institutional Review Board of Asan Medical Center, and informed consent was waived because of the retrospective nature of the study. For planning purposes, 4DCT images were acquired using a CT simulator (LightSpeed RT 16, GE healthcare, Waukesha, WI) during free breathing; during image acquisition, the patients’ respiratory data were recorded using a real-time position management system (RPM, Varian medical systems, Mountain View, CA). The 4DCT images were then synchronized with the respiratory data and sorted by respiratory phase into 10 bins. The entire 10-bin data were transferred to the Varian Eclipse treatment planning system for contouring and planning.

The gross tumor volume (GTV) was delineated on the end-expiratory phase CT image. No clinical target volume (CTV) was defined. For gated treatment, each directional tumor motion from the end-expiratory position was measured from the 10-phase 4DCT images either for full respiratory phases or for the respiratory gating window of 30%–70%. The final decision for gated treatment was made by accounting for the degree of internal target volume (ITV) margin reduction, breathing regularity, and proximity to critical structures. The measured non-isotropic 3D tumor motion was added to the GTV to define the ITV for non-gated or gated radiotherapy. The ITV was expanded with an isotropic 5-mm margin, resulting in a planning target volume (PTV) that accounted for the uncertainties associated with target definitions and inter- and intra-fraction variations. Organs at risk (OARs), including the lungs, spinal cord, esophagus, and heart, were also contoured.

Tumor characteristics are summarized in Table 1. The PTVs of the 22 cases ranged from 4.8 to 40.3 cm^3^. The tumor motion ranged between 0.1 and 1.9 cm. Taking into account the range of tumor motion, regularity of breathing, and proximity to critical structures, respiratory-gated SBRT [13] with a reduced motion range of 0.3–0.8 cm after gating was selected for treatment in 10 out of the 22 cases.

**Table 1:** Tumor characteristics with and without gating.

Case No.	PTV (cm^3^)	GTV (cm^3^)	Tumor Location	Tumor motion Max. range (cm)	Tumor motion Gated range (cm)
1	40.3	12.4	RUL	0.8	-
2	10.3	1.7	RML	1.3	0.4
3	20.3	4.3	RML	0.7	-
4	8.1	1.1	RUL	0.5	-
5	10.9	1.7	LLL	1.9	0.5
6	14.9	2.8	LUL	0.6	0.3
7	8.4	0.9	RUL	0.7	-
8	5.1	0.3	RUL	0.6	-
9	4.8	0.4	RUL	0.4	-
10	5.8	0.4	RML	1.0	0.3
11	10.8	1.2	RUL	0.7	-
12	5.3	0.3	LLL	1.9	0.5
13	7.1	1.0	LUL	1.9	0.3
14	15.5	3.4	LUL	0.1	-
15	6.9	1.1	LLL	0.7	-
16	12.3	1.6	RLL	1.7	0.8
17	10.7	1.5	LLL	1.8	0.6
18	9.4	1.5	RUL	0.7	0.3
19	13.7	2.5	LUL	0.6	0.5
20	12.8	1.8	RLL	1.0	-
21	7.1	0.9	LUL	0.4	-
22	7.8	0.6	RUL	0.7	-

*Abbreviations*: PTV, planning target volume; LLL, left lower lobe; RLL, right lower lobe; RML, right middle lobe; LUL, left upper lobe; RUL, right upper lobe.

### 2. VMAT plan

With the delineated target and OAR volumes on the exhale CT data, VMAT plans were generated using inverse optimization of the Eclipse treatment planning system. Dose was calculated using the Eclipse anisotropic analytical algorithm (AAA) with correction for tissue heterogeneity. All plans employed the use of flattening-filter-free 6 MV photon beams of a Varian Truebeam linear accelerator [14]. The VMAT plans utilized two either half or full-rotating coplanar arcs to minimize dose delivery at the contralateral lung.

The prescribed doses for the PTV were 60 Gy in 4 fractions for ten patients and 60 Gy in 5 fractions for twelve patients. The following planning objectives for the PTV, based on the Radiation Therapy Oncology Group (RTOG) lung SBRT protocols [15–17], were adopted: 1) 95% of the PTV to receive the prescribed dose whilst maintaining hot spots within the PTV; 2) 99% of the PTV to receive >90% of the prescribed dose, and 3) the conformity index (the ratio of the total volume that receives 100% of the prescribed dose to the PTV) to be <1.2.

Additional planning objectives for highly compact dose distribution were as follows: 1) high dose spillage, defined as the amount of normal tissue that receives 105% of the prescription dose, should not exceed 15% of the PTV volume; 2) maximum dose to normal tissue in 2.0 cm in all directions from the PTV (D_2cm_) should not exceed 50%–77% of the prescribed dose, depending on the size of the PTV; and 3) R_50%_ (the ratio of the volume that receives 50% of the prescribed dose to the PTV) was not to exceed 2.9–5.9, depending on the size of the PTV. As for the dose–volume constraints for OARs, the target volume of the total lung that received 20 Gy (V_20_) was <10%. For other OARs, such as the spinal cord, heart, and esophagus, the maximum point dose limitations recommended in the RTOG protocols were adopted.

### 3. Dose rate modulated arc therapy (DrMAT)

**Fig 1:**
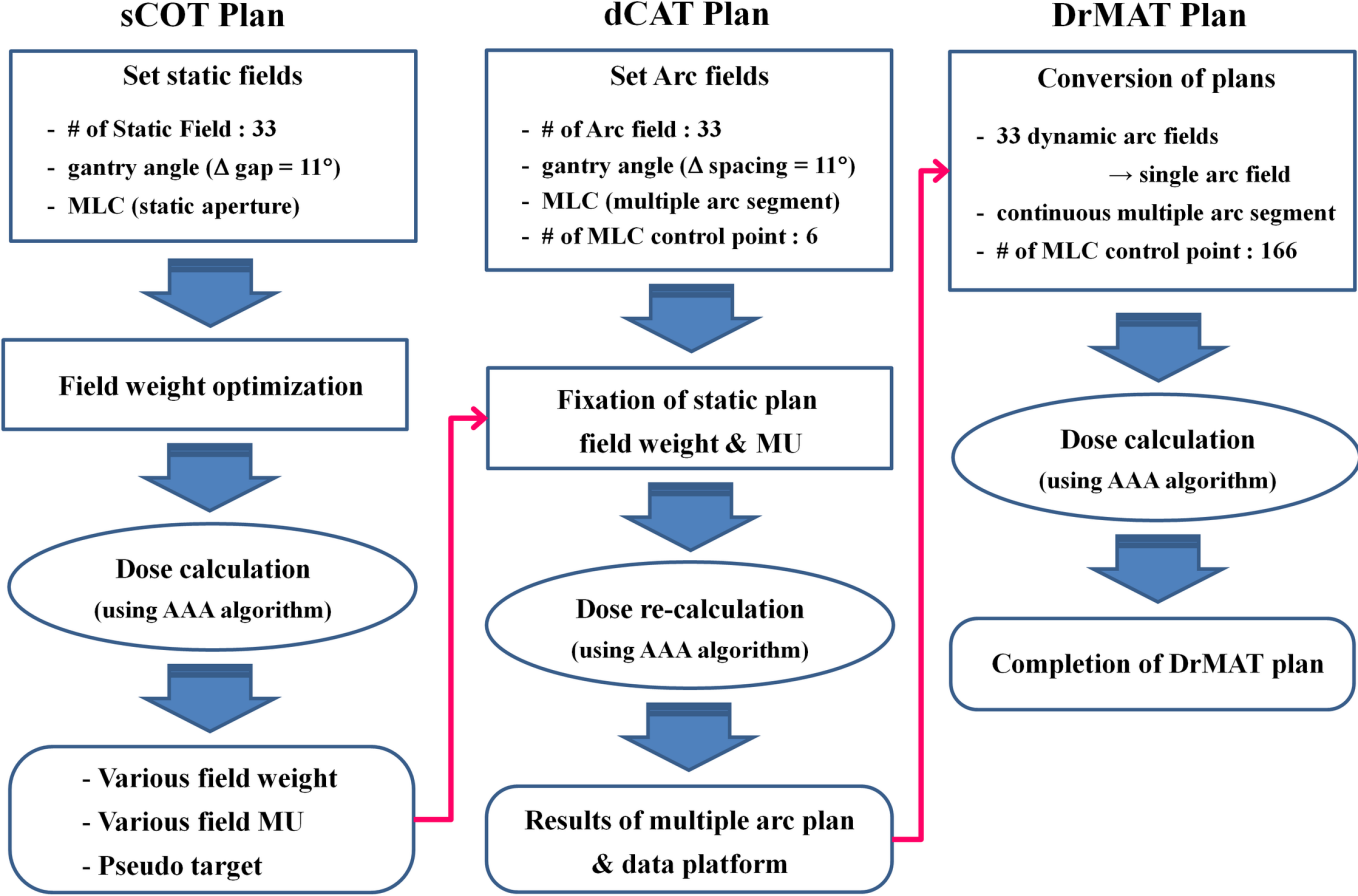
Development process for a dose rate modulated arc therapy (DrMAT) plan via intermediate stages of a static conformal therapy (sCOT) plan and a dynamic conformal arc therapy (dCAT) plan. *Abbreviations*: AAA, anisotropic analytical algorithm; MU, monitor unit.

A schematic illustration of the steps in plan development is presented in Fig 1. DrMAT plans were retrospectively generated and consisted of three main steps: 1) the generation of multiple static beams for a static conformal therapy (sCOT) plan and optimization of their field weights, 2) the conversion of each static beam of the sCOT plan into an arc beam for a dynamic conformal arc therapy (dCAT) plan, and 3) merging all dCAT arc beams into a single DrMAT beam. Each of the procedures is described in detail below.

#### 1) sCOT (static conformal therapy)

**Fig 2:**
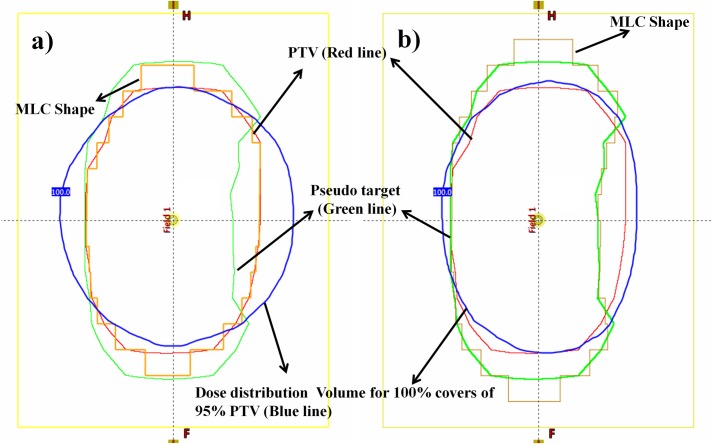
Virtual PTV, a pseudo target for an optimal field aperture: a) the PTV (red) and the isodose volume (blue) that covered 95% of PTV when each field aperture was set such that MLC (yellow) was tightly fitted to the PTV in its beam’s-eye view; b) the isodose volume (blue) that covered 95% of PTV after MLC (yellow) of each field was readjusted to fit the shape of the virtual PTV in its beam’s-eye view. *Abbreviations*: PTV, planning target volume; MLC, multileaf collimator.

A sCOT plan consisting of 33 static fields located 11° apart from each other and covering approximately full gantry rotation was created. MLC leaves of each field were initially positioned such that its aperture coincided with the shape of the PTV in the beam’s eye view (Fig 2). Collimator angle of each static field in the sCOT plan was fixed at 0°. Given the planning objectives for the PTV and OARs the optimal beam weights for each field were determined in the Eclipse inverse planning environment using a field weight optimization algorithm [18]. Unlike full IMRT optimization, field weight optimization modifies the beam weights only without the creation of an optimal fluence map. This option is available for open fields and fields that contain blocks or static MLCs. Since it only supports a pencil beam dose calculation algorithm (PBC) of Eclipse, the dose distribution was re-calculated using the AAA algorithm after beam weight optimization.

Since the MLC of each beam of the sCOT plan was initially set to tightly fit the PTV, the dose coverage of the PTV was not enough in the superior-inferior (SI) direction due to an insufficient penumbral margin (Fig 2). In the axial direction, the same dose volume was spilled over the PTV because of the coplanar arrangement of the sCOT beams, and its distribution was dependent on their beam weights. To identify the optimal penumbral margin around the PTV, the distance between the PTV and the isodose volume that covered 95% of the PTV (supposed to be the prescribed dose) was measured in each left-right, anterior-posterior, and superior-inferior direction. Subsequently, a pseudo target, i.e., a virtual PTV was created from the PTV by addition or subtraction of the distance in each direction. Finally, the MLC shape of each beam initially fitted to the PTV was readjusted to fit the virtual PTV.

#### 2) dCAT (dynamic conformal arc therapy) plan

After achieving optimal field weights for the 33 sCOT beams, each static field was manually converted into a dynamic arc beam with its MLC aperture and beam weight (equivalent to the corresponding MUs). Each converted arc beam covered gantry rotation range of ± 5.5° from the gantry angle of the static beam to fill the gantry rotation space between the adjacent static beams of the sCOT plan. At this time, a static MLC was converted into an arc segment consisting of 6 MLC control points, such that the aperture shape of the MLC changed according to the shape of the virtual PTV at every 2.2° in the gantry angle. Thus, a multiple arc plan, named the dynamic conformal arc therapy (dCAT) plan, which consisted of 33 contiguous arc fields with the gantry rotating at every 11° was generated.

**Fig 3:**
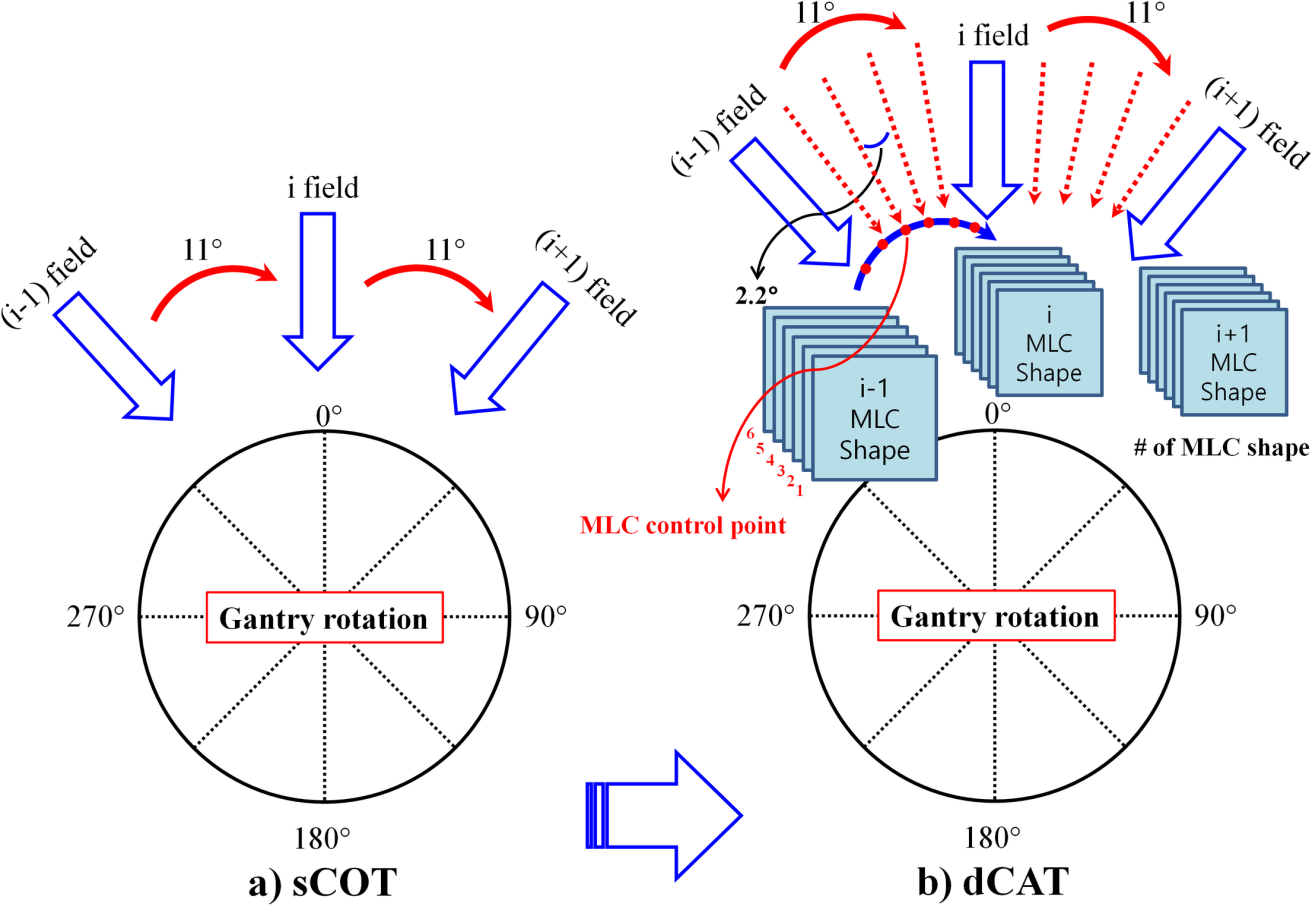
Schematic illustration of the procedure to transform (a) a static conformal therapy (sCOT) plan into (b) a dynamic conformal arc therapy (dCAT) plan. *Abbreviations*: PTV, planning target volume; MLC, multileaf collimator.

A schematic illustration of the procedure used to transform each static beam of the sCOT plan into a sub arc beam of the dCAT plan is presented in Fig 3.

#### 3) DrMAT (dose rate modulated arc therapy) plan

Finally, the dCAT plan composed of 33 adjoining arc beams was merged into a single arc VMAT beam, referred to as the dose rate modulated arc therapy (DrMAT) plan. The DrMAT beam is a single arc plan comprising 166 total control points, less than 177 (the maximum number of control points for a single arc in Varian VMAT), that change the dose rate and the shape of MLC at every 2.2° in gantry rotation.

For this purpose, a DICOM plan file of the dCAT was exported from Eclipse, modified into DrMAT using an in-house Matlab program (MathWorks, Natick, MA, USA), and then re-imported into Eclipse for dose calculations.

The DrMAT plan created is a single arc VMAT plan that does not involve fluence modulation, but delivers an optimal dose rate and field shaping *vs*. gantry angle modulation. Safe and efficient delivery of the DrMAT plan was then automatically determined via the Eclipse planning system taking into account the specified technical limits of the treatment machine including the maximum allowed dose rate, the speed and acceleration of gantry rotation and the maximum speed of leaf motion.

### 4. Plan quality

The plan quality of DrMAT was compared with that of VMAT using the following dosimetric parameters for the target and OARs: 1) conformity index (CI) defined as the ratio of prescription isodose volume to the PTV [19]; 2) D_2cm_, the maximum dose at 2 cm from the PTV in any direction as % of the prescribed dose (PD), D_2cm_ (Gy) = % × PD; 3) R_50%_, the ratio of 50% isodose volume of the prescribed dose to the PTV; 4) homogeneity index (HI) defined as the ratio of the maximum PTV dose to the prescribed dose; 5) the maximum point dose to the spinal cord, esophagus, and heart; and 6) V_20_ of normal lung following the aforementioned RTOG protocols.

Paired *t*-tests were used to compare CI, HI, and D_2cm_ while the Wilcoxon signed rank test was used for R_50%_ as the distribution of R_50%_ did not qualify the normality test. Similarly, the paired *t*-test was used to compare the maximum point dose to esophagus, while Wilcoxon signed rank tests were used for V_20_, and the maximum point doses to the spinal cord and heart.

### 5. Delivery accuracy

The DrMAT plans were delivered using a clinical linac to verify the feasibility of its clinical application in the 10 cases treated by respiratory-gated VMAT. To evaluate the impact of respiratory motion on the dosimetric accuracy of the actual treatment, both the DrMAT and VMAT plans were delivered on a one-dimensional moving phantom (QUASAR™, Modus Medical Devices Inc., London, Canada) under two different delivery scenarios; static and gated delivery. During the gated beam delivery for each case, the moving phantom was controlled in the longitudinal direction by the respiratory signal recorded during 4DCT acquisition of the same patient. The magnitude of the phantom movement that represented the tumor motion was set such that the amplitude of the respiratory signal produced the full range of tumor motion measured from 4DCT data.

The delivered dose distribution was measured using EBT3 films with an Epson 10000XL scanner through a FilmQA Pro software (Ashland Advanced Materials, Wayne, NJ, USA) followed by the procedure described by Lewis *et al* [20, 21]. All irradiated film images were converted to a dose map using a calibration response curve measured during the experiment. The measured dose distribution was compared with the calculated dose distributions via gamma analysis either with dose tolerance of 2% within 2 mm or of 2% within 1 mm. The gamma criteria for SBRT selected in this study were more stringent and demanded a higher accuracy as compared to that used for conventional IMRT, where 3%/3mm is generally accepted.

## Results

### 1. Plan quality

**Fig 4:**
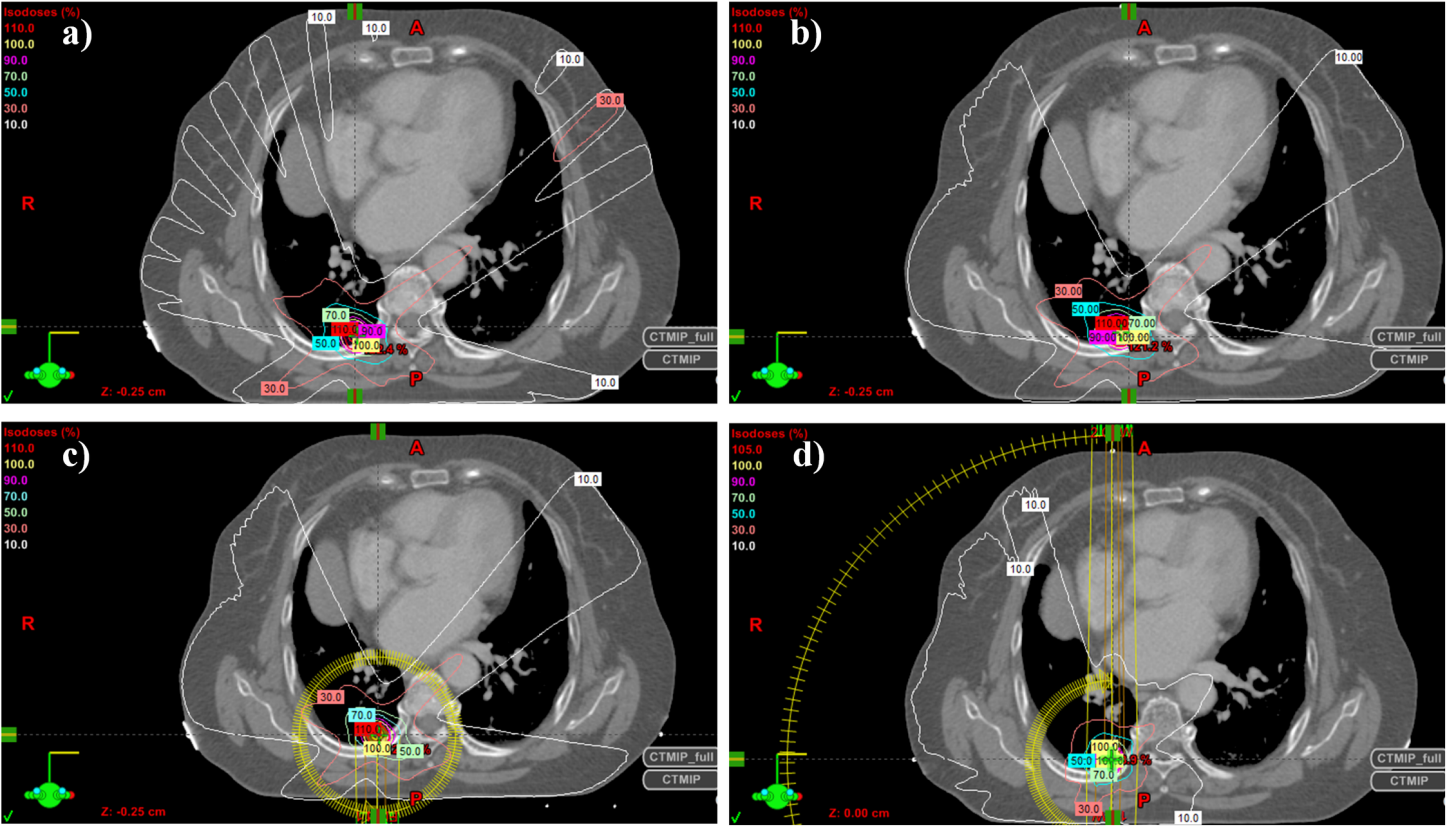
Example of dose distributions for Case no. 9: a) static conformal therapy (sCOT) plan, b) dynamic conformal arc therapy (dCAT) plan, c) dose–rate modulated arc therapy (DrMAT) plan, and d) volumetric arc therapy (VMAT) plan.

Fig 4 shows a typical example of the isodose distribution of each intermediate stage plan (sCOT and dCAT), the final DrMAT, and the original VMAT plan. As expected, in low dose region the dose distribution of dCAT is smoother than that of sCOT because of the transition from discrete static beams to continuous arc beams, while it is the same as that of the DrMAT.

**Fig 5:**
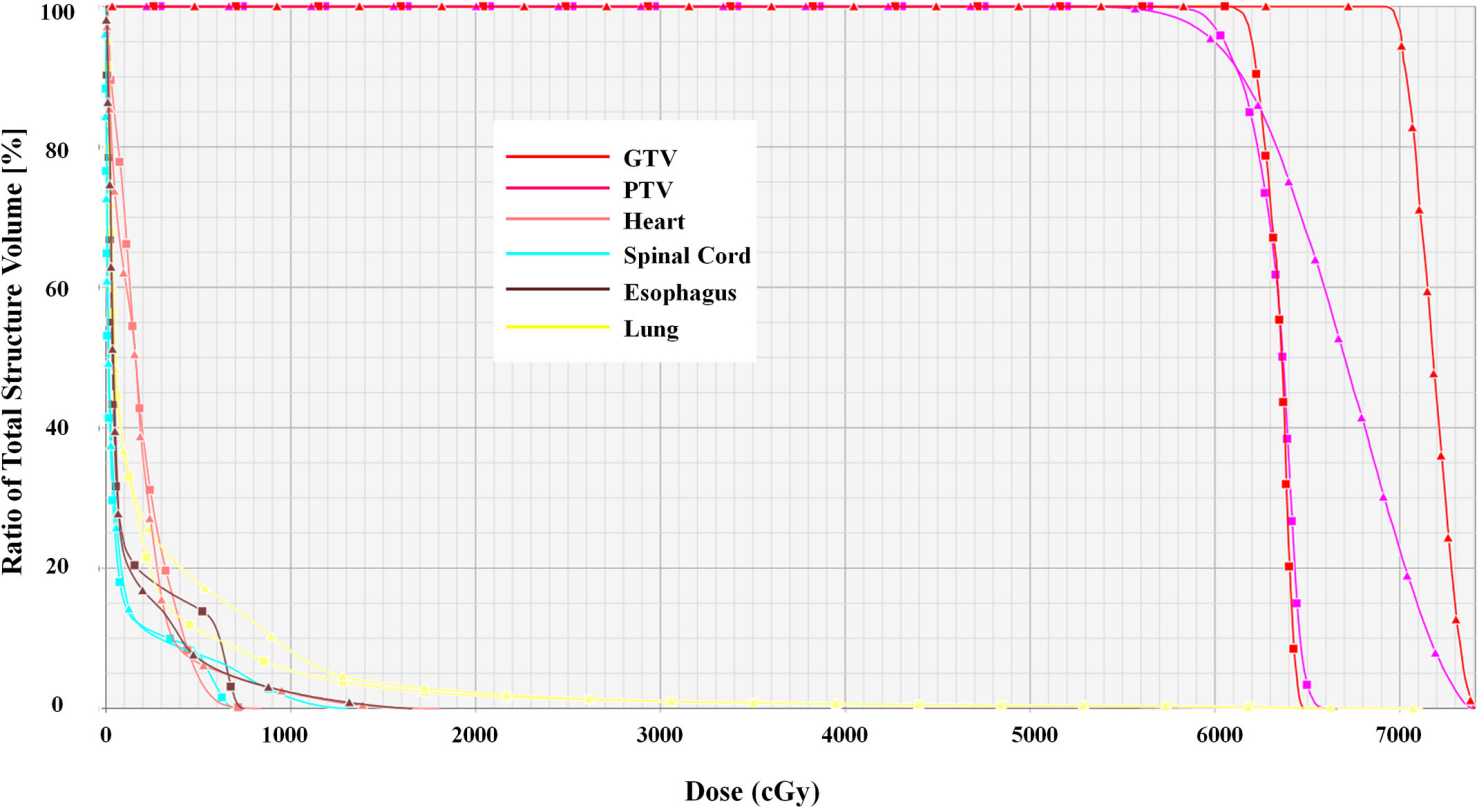
Comparison of dose-volume histograms between DrMAT (▲) and VMAT (■) plans for the same Case no. 9. *Abbreviations*: GTV, gross tumor volume; PTV, planning target volume.

Dose–volume histograms (DVHs) of the same case show that although the DrMAT plan had the similar PTV coverage as that of the VMAT plan, i.e., 95% of the PTV received 60 Gy of the prescribed dose, it had much higher hot spots in the GTV and PTV as compared to those in VMAT (Fig 5). From an SBRT perspective of tumor ablation, however, the hotspots of DrMAT, which were caused by the removal of intensity modulation from VMAT that improved dose homogeneity inside the tumor, are not clinically meaningful as long as they are well confined within the tumor. As for OAR dose sparing, although both the plans satisfied the dose constraints of the RTOG lung SBRT protocols, a slightly better reduction in the OAR doses was achieved with the VMAT plan vis-a-vis the DrMAT plan.

There was no significant difference between the DrMAT and VMAT plans with respect to the CI, D_2cm_, R_50%_, and lung V_20_, which indicates a comparable plan quality with respect to target conformity and dose compactness around the target volume, while both plans also meet the criteria of the RTOG protocols (Table 2).

**Table 2:** Dosimetric characteristics of the VMAT and DrMAT plans.

	VMAT (Mean ± SD)	DrMAT (Mean ± SD)	*p* value
CI	1.03 ± 0.03	1.04 ± 0.03	0.287
D_2cm_ (Gy)	45.7 ± 4.7	44.0 ± 5.8	0.135
R_50%_	4.64 ± 0.46	4.58 ± 0.67	0.733
HI	1.11 ± 0.02	1.29 ± 0.09	< 0.001
D_GTV_ (Gy)	64.4 ± 1.0	73.6 ± 4.4	<0.001
D_(PTV-GTV)_ (Gy)	63.4 ± 0.4	66.4 ± 2.2	<0.001
MUs	3505 ± 610	2879 ± 397	< 0.001

*Abbreviations*: CI, Conformity index; D_2cm_, Maximum dose at 2cm from PTV in any direction as % of prescribed dose (PD); R_50%_, Ratio of 50% isodose volume to the PTV; HI, Homogeneity index (= maximum PTV dose/prescribed dose); MUs, Monitor units

The maximum PTV dose of the DrMAT was significantly higher than that of the VMAT by approximately 20%, which led to a much higher HI, because of a lack of intensity modulation (as already explained).

With regard to the increased hotspots due to DrMAT as opposed to VMAT, mean doses of the GTV and the PTV-GTV, averaged over the 22 patients, were increased by 14.3% and 4.7%, respectively. The total MUs of the DrMAT plan were smaller than those of the VMAT plan by about 20%. The ability to shorten the treatment time is an important advantage of the DrMAT in such a time intensive treatment like SBRT.

With respect to lung V_20_, both the VMAT and DrMAT plans appear to be similar and far below the dose limits of the RTOG protocols. This may be caused by the small GTV size in this study and the reduced ITV margin with motion management of respiratory-gated treatment. Small-sized lung tumors (2–4 cm in length) are frequently encountered at our institution due to the availability of advanced diagnostic modalities and the frequent screening of suspected patients.

However, the maximum point dose to the spinal cord, heart, and esophagus for the DrMAT plans are significantly higher than those of the VMAT plans, but still well below the dose constraints of the RTOG protocols (Table 3).

**Table 3:** Comparison of critical structure doses between the VAMT and DrMAT plans.

Critical structures	RTOG protocol	VMAT	DrMAT	*p* value
Lung V_20_	< 10.0	3.0 (1.4–6.3)	2.9 (1.4–5.7)	0.936
Max. point dose (Gy): Spinal Cord	21.9	6.9 (3.5–11.9)	10.1 (3.3–15.6)	< 0.001
Max. point dose (Gy): Heart	30.0	8.5 (0.3–21.7)	10.1 (0.3–25.7)	0.004
Max. point dose (Gy): Esophagus	25.2	7.6 (4.0–10.9)	11.9 (6.4–15.6)	< 0.001

*Abbreviations*: RTOG, Radiation Therapy Oncology Group; VMAT, volumetric arc radiation therapy; DrMAT, dose rate modulated arc therapy. Data are presented mean values (range) (N = 22).

### 2. Delivery Accuracy

The DrMAT plan was successfully delivered using a Truebeam linac without any interlocks, which demonstrates that the machine parameters for delivering the simplified VMAT plan are well within the limits specified for the machine.

Control point is a building block for all dynamic treatments, where a number of machine parameters can be varied. During beam delivery, all the machine parameters are checked every 10 or 50 ms to ensure that the obtained values are the same as the planned values at the delivered MUs, and if they are not equal, beam is held off until they are all at the right positions. The delivery of the DrMAT plan was accomplished by the sequential execution of 166 control points each of which specified MLC leaf positions, dose rate, and gantry rotation speed as a function of MUs.

**Fig 6:**
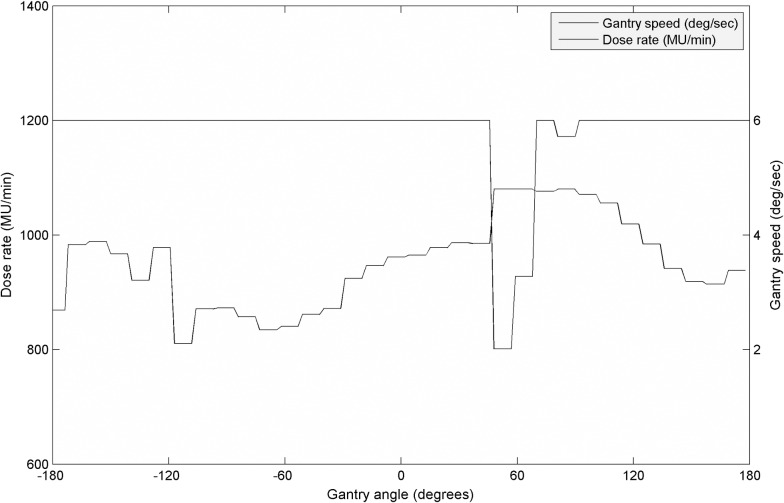
Variations of dose rate and gantry speed as a function of gantry angle, which are executed using a total of 166 control points for a DrMAT plan. Note that every 5 control points at 2.2° intervals have the same values because an 11° sub-arc of the dCAT is equally divided by them.

Fig 6 shows one example of the DrMAT plan presenting modulation of dose rate and gantry speed as a function of gantry angle implemented by 166 control points with 2.2° intervals.

For the Truebeam machine used in the study, the maximum dose rate and gantry speed were set to 1200 MU/min and 4.8 deg/s, respectively. To maximize the delivery efficiency, i.e., minimize the delivery time, either the dose rate or gantry speed was chosen as the maximum value.

With the maximum dose rate and gantry speed, the maximum MUs for a DrMAT control point (2.2°) was 9.2 MU. If MUs of a certain DrMAT control point were larger than 9.2 MU, the maximum value of 1200 MU/min was chosen for the dose rate while the gantry speed was variably slowed down according to the required MUs. In contrast, if MUs of a certain control were smaller than 9.2 MU, the gantry speed was set at the maximum speed of 4.8 deg/s while the dose rate was decreased accordingly. There were 3 control points for which MUs were less than 9.2 MUs in the case of Fig 6. Averaged over the 22 cases, the modulation ranges of the dose rates and gantry angles were 471 ± 479 MU/min and 2.9 ± 0.8 deg/s, respectively.

**Fig 7:**
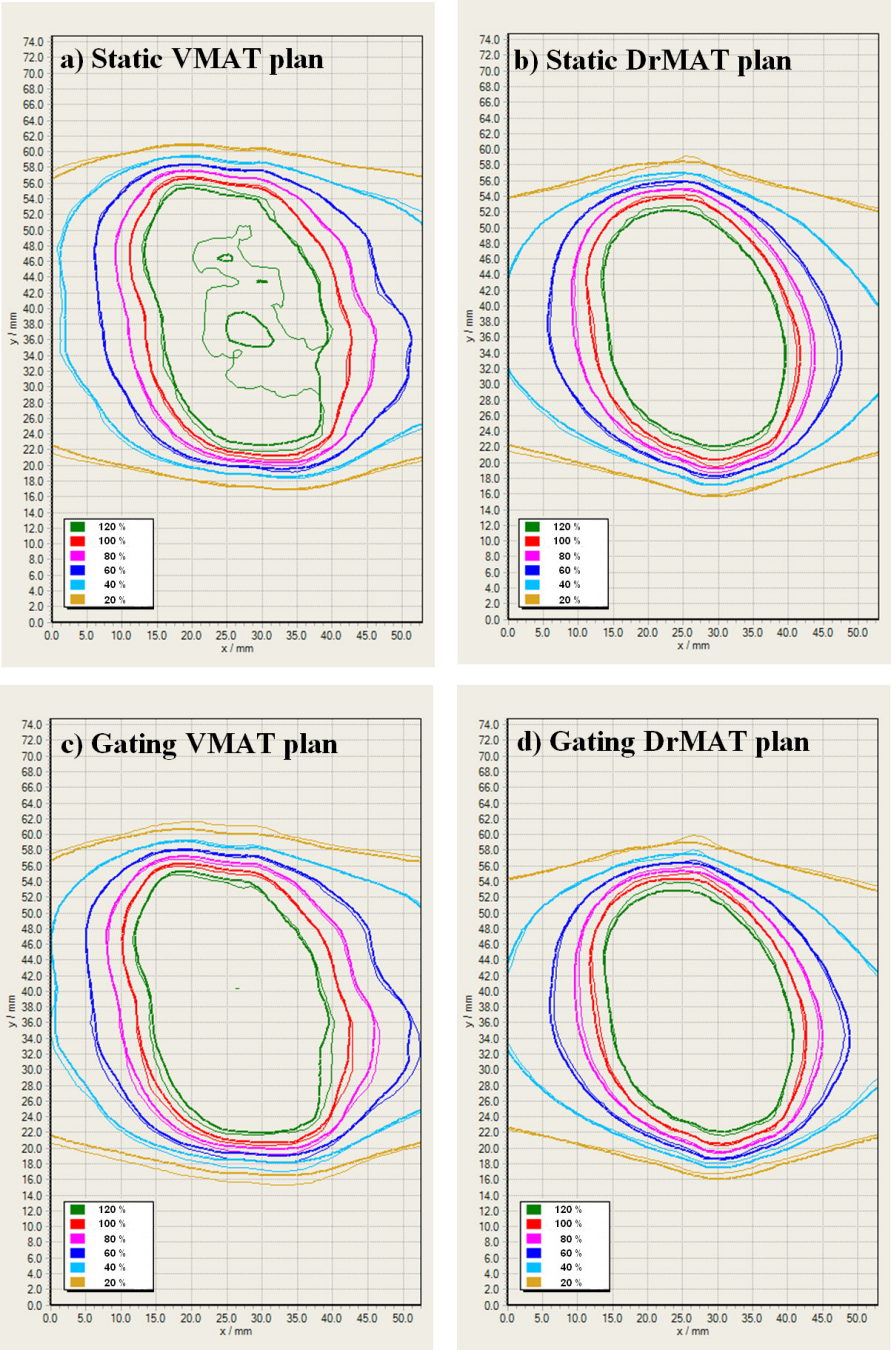
Comparison between the planned and delivered dose distribution for DrMAT and VMAT plans of Case no. 6: a) VMAT plan for static delivery, b) DrMAT plan for static delivery, c) VMAT plan for gating delivery, d) DrMAT plan for gating delivery. The thick and thin lines represent the plan and delivered isodose levels normalized to the prescribed dose, respectively. Note that apparent difference in the dose distributions (e.g., the 100% area) is because they are recalculated ones on a motion phantom, which is different with the patient in the whole size, isocenter location, and density distribution.

As can be seen in Fig 7, compared with the gated DrMAT case, the delivered isodose lines in the gated VMAT case are slightly broader than the planned isodose lines. This is observed for both the target region as well as the surrounding dose gradient region and can be explained by the following facts. First, within the target, VMAT employed an MLC fluence modulation by sophisticatedly patching the borders between multiple field segments to improve the dose uniformity within the target, presuming no tumor motion. However, this does not work when respiratory tumor motion is involved. Second, in the dose gradient region, VMAT employed an MLC fluence modulation to achieve the highest dose gradient around the tumor, again by sophisticatedly patching the borders between multiple field segments, presuming no organ motion. However, respiratory motion degrades the sharp dose gradient and broadens the isodose lines compared with the planned ones.

Fig 7 shows one example of the resulting dose maps of the VMAT and DrMAT plans delivered under two different scenarios (static or gating delivery) were compared with the corresponding calculated dose maps. The performance of the VMAT and DrMAT plans for static delivery is similar. However, the DrMAT plan is superior to the VMAT plan for gating delivery, which is attributable to the absence of the interplay effect between MLC movement and tumor motion with DrMAT.

In the case of static delivery, both VMAT and DrMAT show similar dose delivery accuracy in gamma evaluation with either 2%/2mm or 2%/1mm criteria (Table 4). On the contrary, in the case of gated delivery the dose delivery accuracy of VMAT sharply drops to 87.5%, dosimetrically unacceptable level of decrease from 2 mm to 1 mm in distance-to-agreement. Considering that these performances are achievable with gated delivery, which effectively limits the tumor motion to within about 5 mm, without appropriate motion management the delivery accuracy of VMAT could more sharply drop to clinically unacceptable levels as increasing tumor motion and intensity modulation despite the agreement level of 2%/2mm.

**Table 4:** Gamma evaluation passing rates for VMAT and DrMAT plans.

Gamma criteria	Static delivery (%)		Gating delivery (%)	
	VMAT	DrMAT	VMAT	DrMAT
2% within 2mm	99.7 ± 0.2	99.6 ± 0.3	98.2 ± 1.2	99.0 ± 1.1
2% within 1mm	96.1 ± 2.2	95.9 ± 2.1	87.5 ± 8.4	93.3 ± 3.6

*Abbreviations*: VMAT, volumetric modulated arc therapy; DrMAT, dose rate modulated arc therapy.

**Fig 8:**
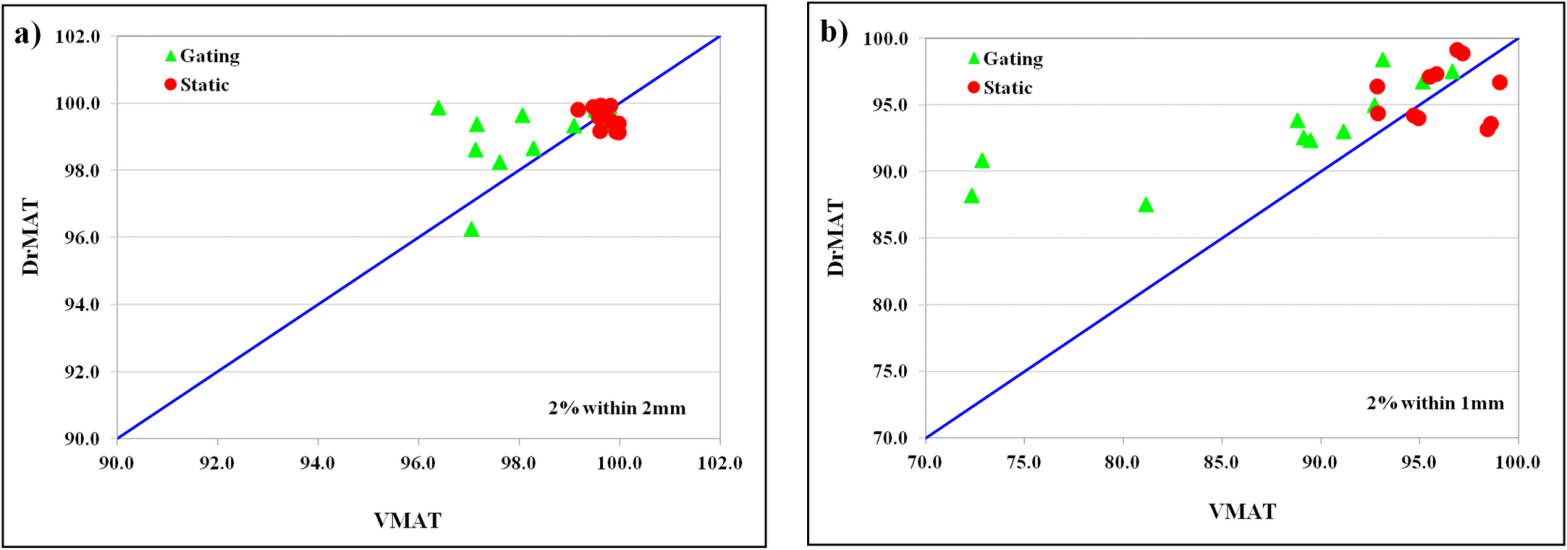
Scatter plots of gamma passing rate between the DrMAT and VMAT plans: a) 2%/2mm and b) 2%/1mm.

Fig 8 shows scatter plots of gamma evaluation for DrMAT and VMAT plans, which demonstrates that the dose delivery accuracy of DrMAT is superior when tumor motion is involved.

## Discussion

A challenge of SBRT is to deliver an ablative dose to the tumor while keeping the dose to the surrounding normal organs under the tolerance dose limit. Therefore, highly conformal radiation dose at the target site and rapid dose fall-off in the surrounding normal tissues is critical for SBRT. To this end, the use of IMRT and VMAT techniques is rapidly growing in SBRT, with superior plan quality in terms of both target dose conformity and critical structure dose sparing [10, 22].

In the majority of lung SBRT cases, however, the target volume is small enough that 3DCRT can generate plans of clinically acceptable quality. In our study cohort, PTV was tiny, ball-shaped, and about 2–4 cm in diameter. Therefore, the dose to the OARs was far below the threshold for severe injury to the lung, esophagus, or spinal cord. In this study, DrMAT resulted in 19%, 57%, and 46% higher maximum point doses to the heart, esophagus, and spinal cord, respectively, compared to those with VMAT. However, this may be caused by the fact that the planning objectives of OARs imposed for inverse optimization were already satisfied. Considering the subtle gain achieved by VMAT, the excessive intensity modulations appear to be unnecessary, or even a source of delivery uncertainty; the choice of IMRT and VMAT should be made wisely and only when more critical structure sparing is necessary.

In an effort to improve dose sparing to OARs whilst retaining the delivery technique as simple as 3DCRT, Ross et al. [23] developed a modified dynamic conformal arc therapy (DCAT) that can be easily implemented on common treatment planning systems. It consists of six coplanar conformal arcs with 59° each and 1° gap between arcs, where beam weights of each arc were manually and repeatedly adjusted to optimize target coverage and dose sparing to OARs. In a study comparing 3DCRT, DCAT, and VMAT [24], VMAT and DCAT improved dose distributions to the PTV, but VMAT was superior to DCAT in terms of both high-dose spillage (CI) and low-dose spillage (D_2cm_ and R_50%_). Moreover, DCAT resulted in 14%, 78%, and 92% higher maximum point doses to the heart, esophagus, and spinal cord, respectively, compared to those with VMAT. They concluded that DCAT planning requires a delicate balance to achieve OAR constraints while not violating high- and low-dose spillage indices.

In this study, we use a far greater number of arcs, i.e., 33 sub arc beams in DrMAT, and achieved field weight optimization through dose–volume based inverse planning process to automatically determine optimal beam weights for each arc component. Furthermore, the use of a pseudo target can solve heterogeneous dose distribution around the tumor caused by the eccentric beam weights determined via the field weights optimization. In this way DrMAT achieves equivalent plan quality both with respect to high-dose and low-dose spillage. Furthermore, when comparing the aforementioned results on dose sparing to OARs between DrMAT and DCAT with respect to VMAT, it appears that dose–volume-based inverse planning of DrMAT is somewhat effective in OAR sparing by figuring out the optimal field weights for 33 sub arcs.

Another alternative approach, referred to as beam-controlled arc therapy (BCAT), has also been investigated [25]. In this approach, the radiation beam is controlled on or off while delivering intensity-modulated arc therapy. By employing linear-programming-based dose optimization to each aperture weight, the radiation beam was held off at control points with zero weights rather than the beam weight modulation via dose rate and gantry speed. By removing unnecessary beam apertures with beam-holds at certain gantry angles BCAT was superior to VMAT in dose sparing to OARs and in dose uniformity for head and neck cancers by up to 17% and 57%, respectively. However, simultaneous use of this method with respiratory-gated treatment is a challenge.

An additional challenge of SBRT in thoracic and abdominal tumors is the respiratory tumor motion. The respiratory gating technique aims to spare the surrounding normal tissue by reducing the component of the PTV to account for respiratory motion. In our study cohort, the free-breathing tumor motion ranged from 0.1 to 1.9 cm. Considering the ranges of tumor motion with and without gating, regularity of breathing, and proximity to critical structures, ten out of the 22 cases were treated with respiratory-gated SBRT with their reduced tumor motion range of 0.3 to 0.8 cm after gating.

The complexity of SBRT, including multiple beams, image guidance procedures and the delivery of a large number of monitor units (MUs), may be more time intensive per fraction than conventionally fractionated radiotherapy [26]. Intrafraction tumor position variations that might compromise the delivered dose accuracy increase with the duration of the treatment session [27]; furthermore long treatment times may not be tolerated by patients [28]. Therefore, shortening the treatment duration is important for SBRT. In this study the dose delivery efficiency of SBRT was improved by 20% using DrMAT compared to that using VMAT.

As described well in the debate article, a major concern with the use of IMRT and VMAT for SBRT lung cancer is whether the motion of the tumor leads to significant dosing discrepancy, especially while delivering a high dose per fraction. As the MLCs move across the field, individual leaves may cover part of the target during treatment delivery. Any target movement will result in the dose not being delivered as planned. The interplay between MLC motion, jaw movement, gantry rotation, and target motion during free-breathing treatment with VMAT is complex when compared to an open field like 3DCRT. Therefore, 3DCRT is expected to have a better agreement in target coverage between the plan and delivered treatment compared with that using IMRT and VMAT, especially when there is large respiratory tumor motion or significant modulation in IMRT or VMAT. Nonetheless, not all lung SBRT cases are best treated with 3DCRT. Lung tumors vary in location, size, motion, and grade. IMRT and VMAT are used only for carefully selected patients that have large tumor sizes, minimal tumor motion, or in whom dose sparing cannot be achieved with 3DCRT owing to the proximity of critical structures. Any slight dosimetric advantages of IMRT/VMAT will be diminished or even worsen in practice due to various uncertainties.

As demonstrated with respect to the delivery accuracy, respiratory-gated DrMAT resulted in significantly higher gamma pass rates of 93.3 ± 3.5% when compared with 87.5 ± 8.4% of VMAT on applying the criterion of 2% within 1 mm. As expected, the dose deviations of VMAT were caused by the interplay effect that is strongly dependent on the speed of the beam aperture relative to the speed of the target motion [29]. Unlike VMAT, in DrMAT the tumor is irradiated as a whole by its motion encompassing open fields with fairly uniform intensity. DrMAT plan is therefore more robust than the VMAT plan when tumor motion during respiration is considered.

Unless respiratory-gated SBRT that reduced the range of tumor motion within 0.5–1.0 cm, tumor motion would increase up to 2–3 cm and cause more severe deviations between the planned and delivered dose distribution for VMAT. In an effort to eliminate the complexity of VMAT associated with variation of dose rate and gantry rotation speed, an alternative approach using constant dose-rate delivery with variable angular spacing has been proposed [30]. In a study of four cancer patients (two head-and-neck, one brain and one prostate cancer), the use of variable angular spacing was shown to implement constant dose-rate VMAT plans in clinics not equipped with the new variable dose-rate-enabled machines with comparable plan quality and treatment efficacy. Current implementation of DrMAT facilitates the variable dose-rate approach of VMAT, but the constant dose-rate approach for DrMAT can be easily implemented to achieve further simplification in dose delivery.

Similar to the recent use of modified DCAT to liver SBRT [31] either with or without flattening-filter-free beams [32], DrMAT is also easily applicable to other abdominal cancers that are particularly associated with respiratory tumor motion such as liver and pancreas cancers.

Having demonstrated the safety and efficacy by measurements using a moving phantom, the DrMAT technique seems to be adapted to routine clinical practice without excessive difficulty. To implement the proposed DrMAT, one needs a treatment planning system that provides beam weight optimization functionality. However, beam weight optimization has been a well-developed function since the 3DCRT era and is still available in most commercial TPS systems to the best of our knowledge. In addition, a DICOM tool is necessary to manipulate DICOM plan data, but the standardized protocol of DICOM RT plan removes the specific machine dependence of a plan. However the technique would ultimately need to be tested with patients treated under a protocol that specifies detailed requirements as follows: appropriate selection criteria; detailed procedures of treatment planning including 4DCT, target volume definitions, and plan evaluation methods; quality assurance procedures and evaluation methodology; and treatment delivery procedures including a moving tumor setup using CBCT or fluoroscopic imaging.

## Conclusion

We developed a novel SBRT technique that achieves a plan quality comparable to that of VMAT and is as simple to implement as 3DCRT, without compromising the plan quality in cases where tumor motion is a significant issue. The proposed technique is compatible with the currently available treatment planning systems and equipment.

## Supporting Information

S1 Fig Scatter plot of conformity index (CI) between the DrMAT and VMAT plans. (TIF)

S2 Fig Scatter plot of D_2cm_ between the DrMAT and VMAT plans. (TIF)

S3 Fig Scatter plot of R_50%_ between the DrMAT and VMAT plans. (TIF)

S4 Fig Scatter plot of PTV D_max_ between the DrMAT and VMAT plans. (TIF)

S5 Fig Scatter plot of V_20_ between the DrMAT and VMAT plans. (TIF)

## References

[1] Timmerman R, Paulus R, Galvin J, Michalski J, Straube W, Bradley J, et al Stereotactic Body Radiation Therapy for Inoperable Early Stage Lung Cancer. Jama-J Am Med Assoc. 2010;303(11):1070–6. 10.1001/jama.2010.261 . PMC2907644 20233825

[2] Fakiris AJ, McGarry RC, Yiannoutsos CT, Papiez L, Williams M, Henderson MA, et al Stereotactic Body Radiation Therapy for Early-Stage Non-Small-Cell Lung Carcinoma: Four-Year Results of a Prospective Phase Ii Study. Int J Radiat Oncol. 2009;75(3):677–82. 10.1016/j.ijrobp.2008.11.042 . 19251380

[3] Kavanagh BD, Schefter TE, Cardenes HR, Stieber VW, Raben D, Timmerman RD, et al Interim analysis of a prospective phase I/II trial of SBRT for liver metastases. Acta oncologica. 2006;45(7):848–55. 10.1080/02841860600904870 . 16982549

[4] Dagoglu RN, Callery M, Moser J, Tseng JF, Kent T, Bullock AJ, et al Stereotactic body radiotherapy (SBRT) reirradiation for recurrent pancreas cancer. J Clin Oncol. 2015;33(3). . 10.7150/jca.13295 PMC4747882 26918041

[5] Ong CL, Verbakel WF, Cuijpers JP, Slotman BJ, Lagerwaard FJ, Senan S. Stereotactic radiotherapy for peripheral lung tumors: a comparison of volumetric modulated arc therapy with 3 other delivery techniques. Radiotherapy and oncology: journal of the European Society for Therapeutic Radiology and Oncology. 2010;97(3):437–42. 10.1016/j.radonc.2010.09.027 . 21074878

[6] Wong JW, Sharpe MB, Jaffray DA, Kini VR, Robertson JM, Stromberg JS, et al The use of active breathing control (ABC) to reduce margin for breathing motion. International journal of radiation oncology, biology, physics. 1999;44(4):911–9. . 10386650 10.1016/s0360-3016(99)00056-5

[7] Bouilhol G, Ayadi M, Rit S, Thengumpallil S, Schaerer J, Vandemeulebroucke J, et al Is abdominal compression useful in lung stereotactic body radiation therapy? A 4DCT and dosimetric lobe-dependent study. Physica medica: PM: an international journal devoted to the applications of physics to medicine and biology: official journal of the Italian Association of Biomedical Physics. 2013;29(4):333–40. 10.1016/j.ejmp.2012.04.006 . 22617761

[8] De La Fuente Herman T, Vlachaki MT, Herman TS, Hibbitts K, Stoner JA, Ahmad S. Stereotactic body radiation therapy (SBRT) and respiratory gating in lung cancer: dosimetric and radiobiological considerations. Journal of applied clinical medical physics / American College of Medical Physics. 2010;11(1):3133 . 20160698 10.1120/jacmp.v11i1.3133 PMC5719765

[9] Merrow CE, Wang IZ, Podgorsak MB. A dosimetric evaluation of VMAT for the treatment of non-small cell lung cancer. Journal of applied clinical medical physics / American College of Medical Physics. 2013;14(1):4110 10.1120/jacmp.v14i1.4110 . 23318374 PMC5714051

[10] Rao M, Wu J, Cao D, Wong T, Mehta V, Shepard D, et al Dosimetric impact of breathing motion in lung stereotactic body radiotherapy treatment using intensity modulated radiotherapy and volumetric modulated arc therapy [corrected]. International journal of radiation oncology, biology, physics. 2012;83(2):e251–6. 10.1016/j.ijrobp.2011.12.001 . 22365622

[11] Cai J, Malhotra HK, Orton CG. A 3D-conformal technique is better than IMRT or VMAT for lung SBRT. Medical physics. 2014;41(4). Artn 040601 10.1118/1.4856175 . 24694118

[12] Kim SS, Song SY, Kwak J, Ahn SD, Kim JH, Lee JS, et al Clinical prognostic factors and grading system for rib fracture following stereotactic body radiation therapy (SBRT) in patients with peripheral lung tumors. Lung Cancer. 2013;79(2):161–6. 10.1016/j.lungcan.2012.10.011 . 23182662

[13] Saito T, Matsuyama T, Toya R, Fukugawa Y, Toyofuku T, Semba A, et al Respiratory gating during stereotactic body radiotherapy for lung cancer reduces tumor position variability. PloS one. 2014;9(11):e112824 10.1371/journal.pone.0112824 25379729 PMC4224502

[14] Huang BT, Lu JY, Lin PX, Chen JZ, Kuang Y, Chen CZ. Comparison of Two RapidArc Delivery Strategies in Stereotactic Body Radiotherapy of Peripheral Lung Cancer with Flattening Filter Free Beams. PloS one. 2015;10(7):e0127501 10.1371/journal.pone.0127501 26131554 PMC4488574

[15] Videtic GM, Hu C, Singh A, Chang JY, Parker W, Olivier K, et al Radiation Therapy Oncology Group (RTOG) Protocol 0915: A Randomized Phase 2 Study Comparing 2 Stereotactic Body Radiation Therapy (SBRT) Schedules for Medically Inoperable Patients With Stage I Peripheral Non-Small Cell Lung Cancer. Int J Radiat Oncol. 2013;87(2):S3–S. .

[16] Bezjak A, Paulus R, Gaspar LE, Timmerman RD, Straube WL, Ryan WF, et al Primary Study Endpoint Analysis for NRG Oncology/RTOG 0813 Trial of Stereotactic Body Radiation Therapy (SBRT) for Centrally Located Non-Small Cell Lung Cancer (NSCLC). Int J Radiat Oncol. 2016;94(1):5–6. .

[17] Timmerman RD, Paulus R, Galvin J, Michalski J, Straube W, Bradley J, et al Stereotactic Body Radiation Therapy for Medically Inoperable Early-stage Lung Cancer Patients: Analysis of RTOG 0236. Int J Radiat Oncol. 2009;75(3):S3–S. .

[18] External Beam Planning Reference Guide: Eclipse version 10.0. Varian medical Systems; 2009.

[19] Feuvret L, Noel G, Mazeron JJ, Bey P. Conformity index: A review. Int J Radiat Oncol. 2006;64(2):333–42. 10.1016/j.ijrobp.2005.09.028 . 16414369

[20] Lewis D, Micke A, Yu X, Chan MF. An efficient protocol for radiochromic film dosimetry combining calibration and measurement in a single scan. Medical physics. 2012;39(10):6339–50. 10.1118/1.4754797 . 23039670 PMC9381144

[21] Marrazzo L, Zani M, Pallotta S, Arilli C, Casati M, Compagnucci A, et al GafChromic() EBT3 films for patient specific IMRT QA using a multichannel approach. Phys Medica. 2015;31(8):1035–42. . 10.1016/j.ejmp.2015.08.010 26429383

[22] Zhang GG, Ku LC, Dilling TJ, Stevens CW, Zhang RR, Li WQ, et al Volumetric modulated arc planning for lung stereotactic body radiotherapy using conventional and unflattened photon beams: a dosimetric comparison with 3D technique. Radiation oncology. 2011;6 Artn 152 10.1186/1748-717x-6-152 . PMC3354344 22070866

[23] Ross CC, Kim JJ, Chen ZJ, Grew DJ, Chang BW, Decker RH. A novel modified dynamic conformal arc technique for treatment of peripheral lung tumors using stereotactic body radiation therapy. Practical radiation oncology. 2011;1(2):126–34. 10.1016/j.prro.2010.11.002 24673926

[24] Rauschenbach BM, Mackowiak L, Malhotra HK. A dosimetric comparison of three-dimensional conformal radiotherapy, volumetric-modulated arc therapy, and dynamic conformal arc therapy in the treatment of non-small cell lung cancer using stereotactic body radiotherapy. Journal of applied clinical medical physics / American College of Medical Physics. 2014;15(5):4898 10.1120/jacmp.v15i5.4898 . 25207575 PMC5711086

[25] Zhang HH, Betzel GT, Yi BY, D'Souza WD. Beam controlled arc therapy—a delivery concept for stationary targets. Physics in medicine and biology. 2013;58(20):7117–29. 10.1088/0031-9155/58/20/7117 . 24052088

[26] Nyman J, Johansson KA, Hulten U. Stereotactic hypofractionated radiotherapy for stage I non-small cell lung cancer—Mature results for medically inoperable patients. Lung Cancer. 2006;51(1):97–103. 10.1016/j.lungcan.2005.08.011 . 16213059

[27] Purdie TG, Bissonnette JP, Franks K, Bezjak A, Payne D, Sie F, et al Cone-beam computed tomography for on-line image guidance of lung stereotactic radiotherapy: Localization, verification, and intrafraction tumor position. Int J Radiat Oncol. 2007;68(1):243–52. 10.1016/j.ijrobp.2006.12.022 . 17331671

[28] Brock J, Bedford J, Partridge M, McDonald F, Ashley S, McNair HA, et al Optimising Stereotactic Body Radiotherapy for Non-small Cell Lung Cancer with Volumetric Intensity-modulated Arc Therapy-A Planning Study. Clinical oncology. 2012;24(1):68–75. 10.1016/j.clon.2011.02.003 . 21396808

[29] Yu CX, Jaffray DA, Wong JW. The effects of intra-fraction organ motion on the delivery of dynamic intensity modulation. Physics in medicine and biology. 1998;43(1):91–104. 10.1088/0031-9155/43/1/006 . 9483625

[30] Tang G, Earl MA, Yu CX. Variable dose rate single-arc IMAT delivered with a constant dose rate and variable angular spacing. Physics in medicine and biology. 2009;54(21):6439–56. 10.1088/0031-9155/54/21/001 . 19820268

[31] Shi C, Chen Y, Fang DX, Iannuzzi C. Application of modified dynamic conformal arc (MDCA) technique on liver stereotactic body radiation therapy (SBRT) planning following RTOG 0438 guideline. Medical dosimetry: official journal of the American Association of Medical Dosimetrists. 2015;40(1):26–31. 10.1016/j.meddos.2014.07.002 . 25172164

[32] Smith A, Kim S, Serago C, Hintenlang K, Ko S, Vallow L, et al Use of Flattening Filter Free Photon Beams for Off-axis Targets in Conformal Arc Stereotactic Body Radiation Therapy. Prog Med Phys. 2014;25(4):288–97.

