# Supplementary figures and images for: Improving Delivery Accuracy of Stereotactic Body Radiotherapy to a Moving Tumor Using Simplified Volumetric Modulated Arc Therapy
Source: PLoS One. 2016 Jun 22;11(6):e0158053. doi: 10.1371/journal.pone.0158053 (PMC4917108; doi:10.1371/journal.pone.0158053)

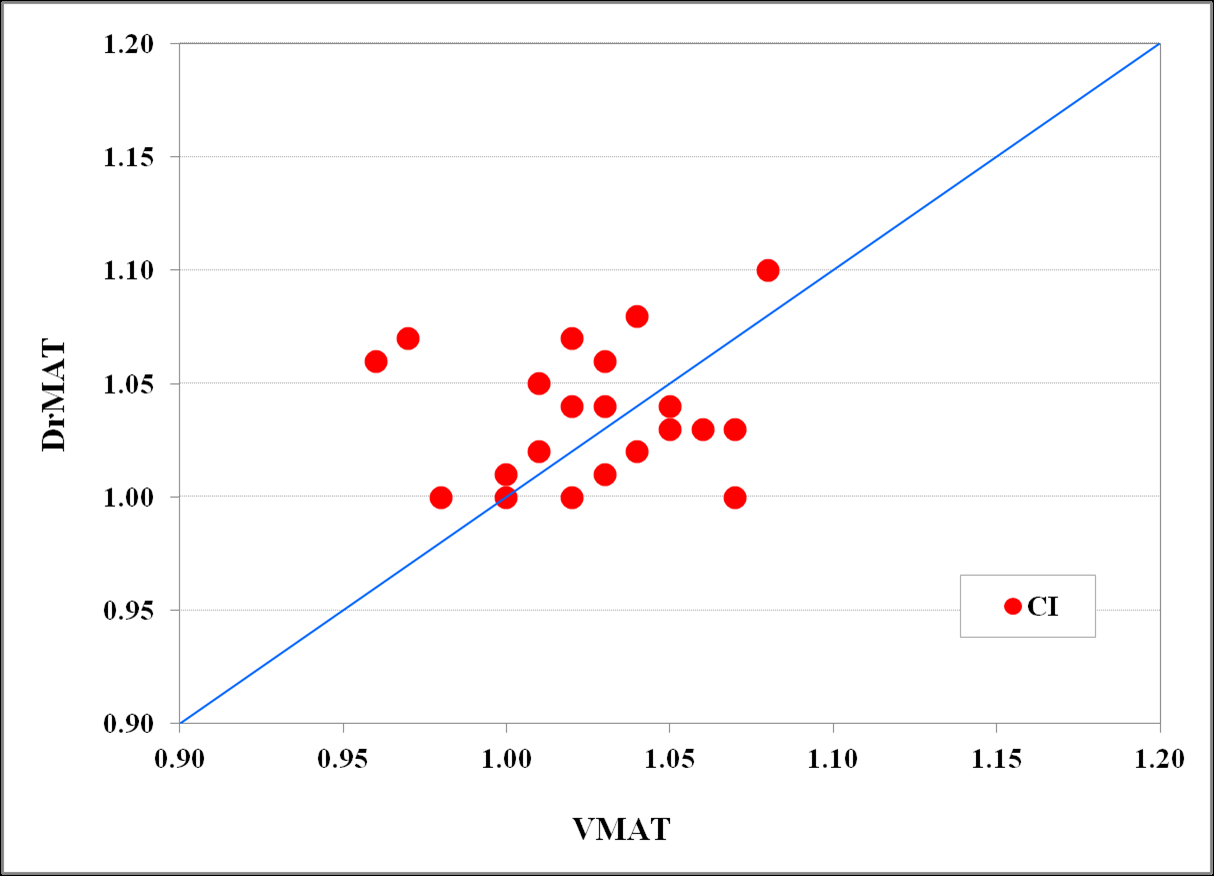

Supplement: S1 Fig — (TIF) [file pone.0158053.s001.tif]

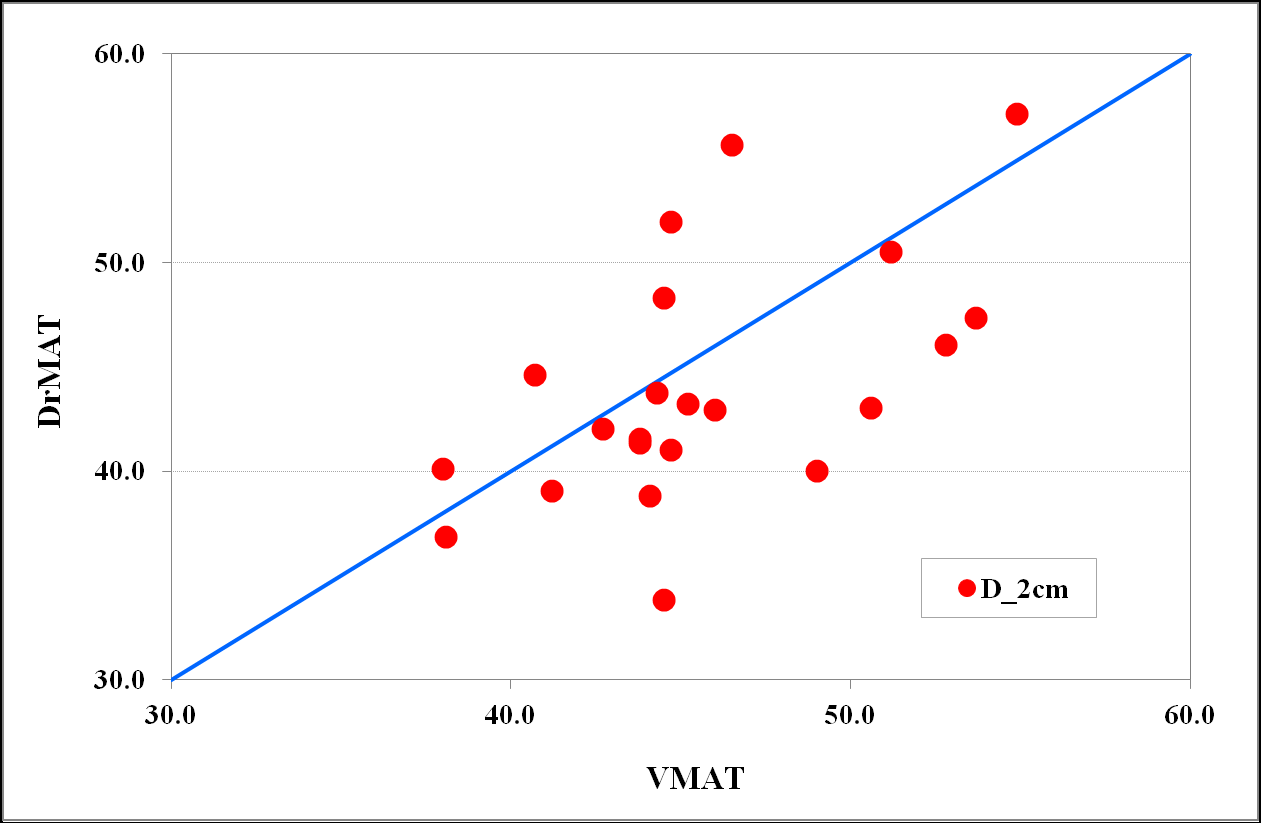

Supplement: S2 Fig — (TIF) [file pone.0158053.s002.tif]

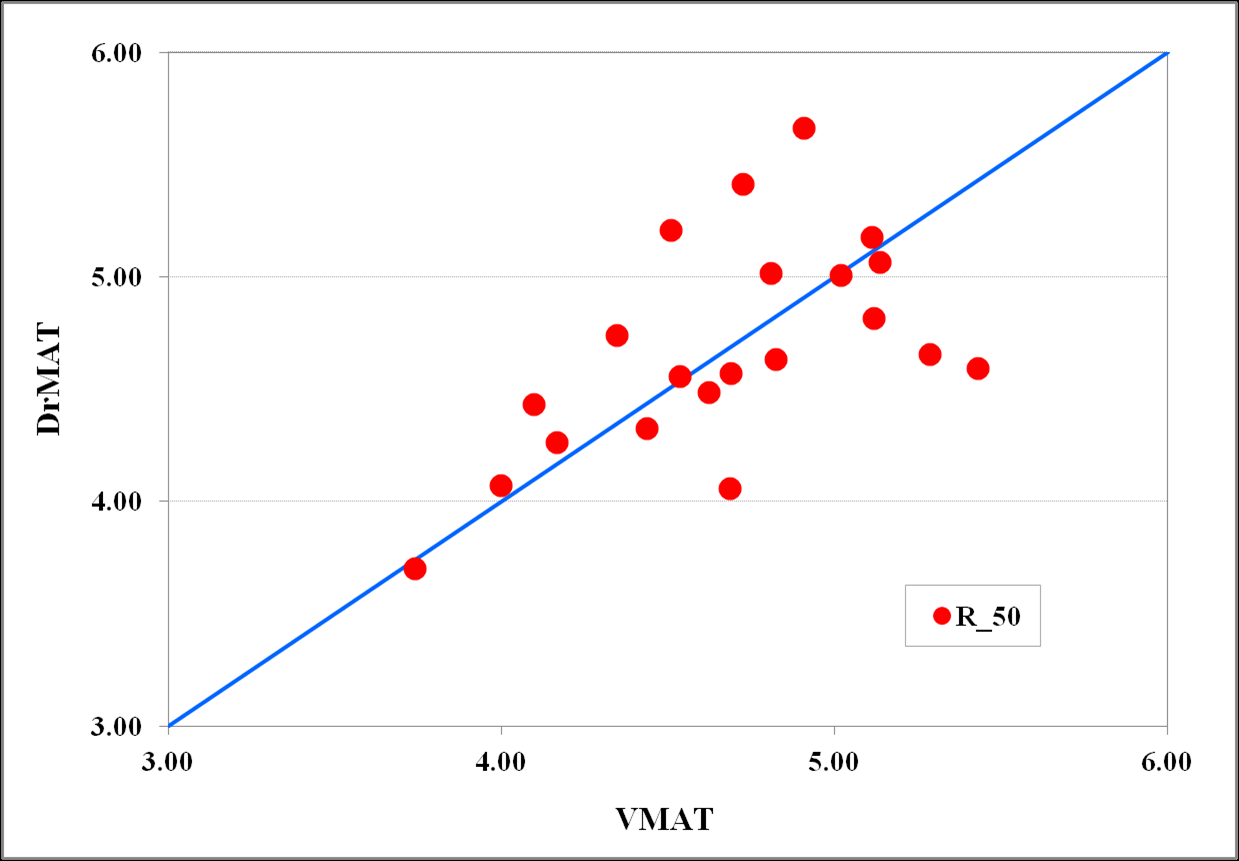

Supplement: S3 Fig — (TIF) [file pone.0158053.s003.tif]

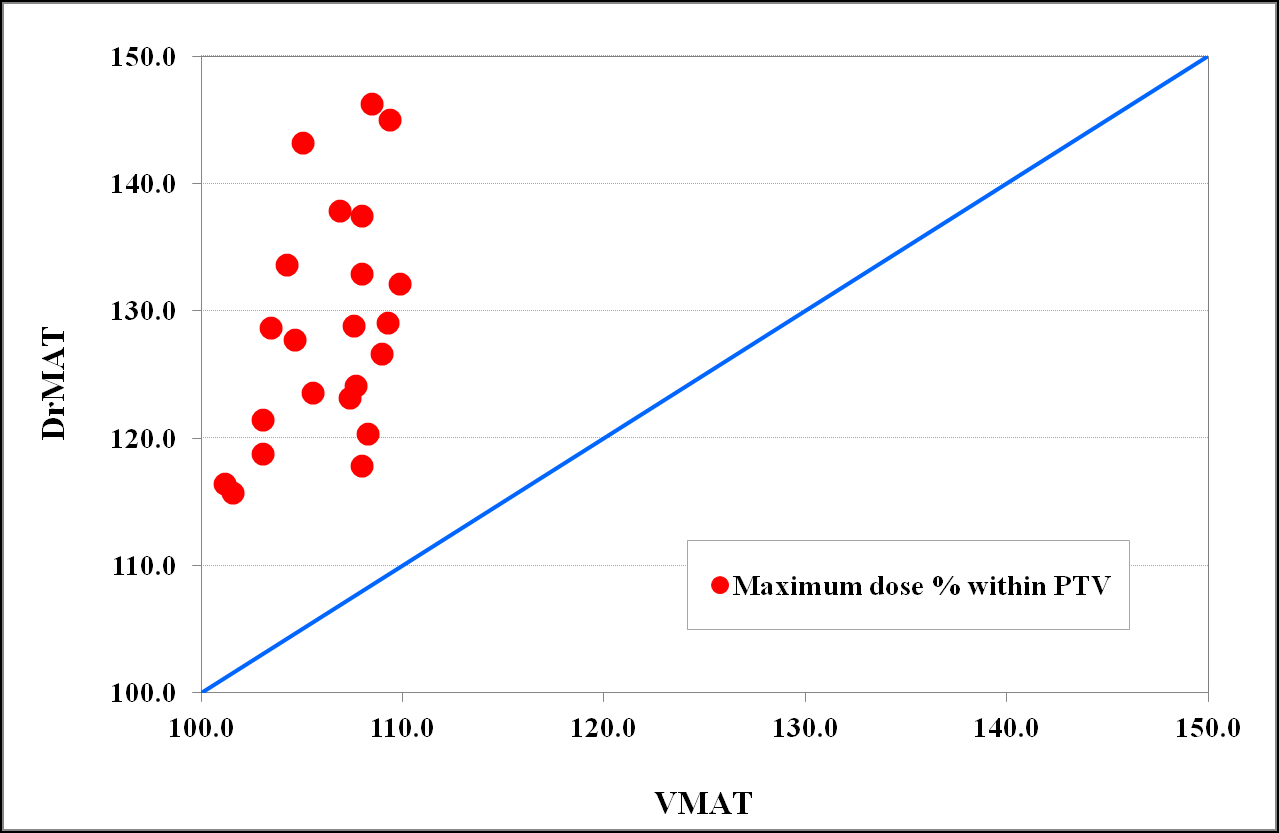

Supplement: S4 Fig — (TIF) [file pone.0158053.s004.tif]

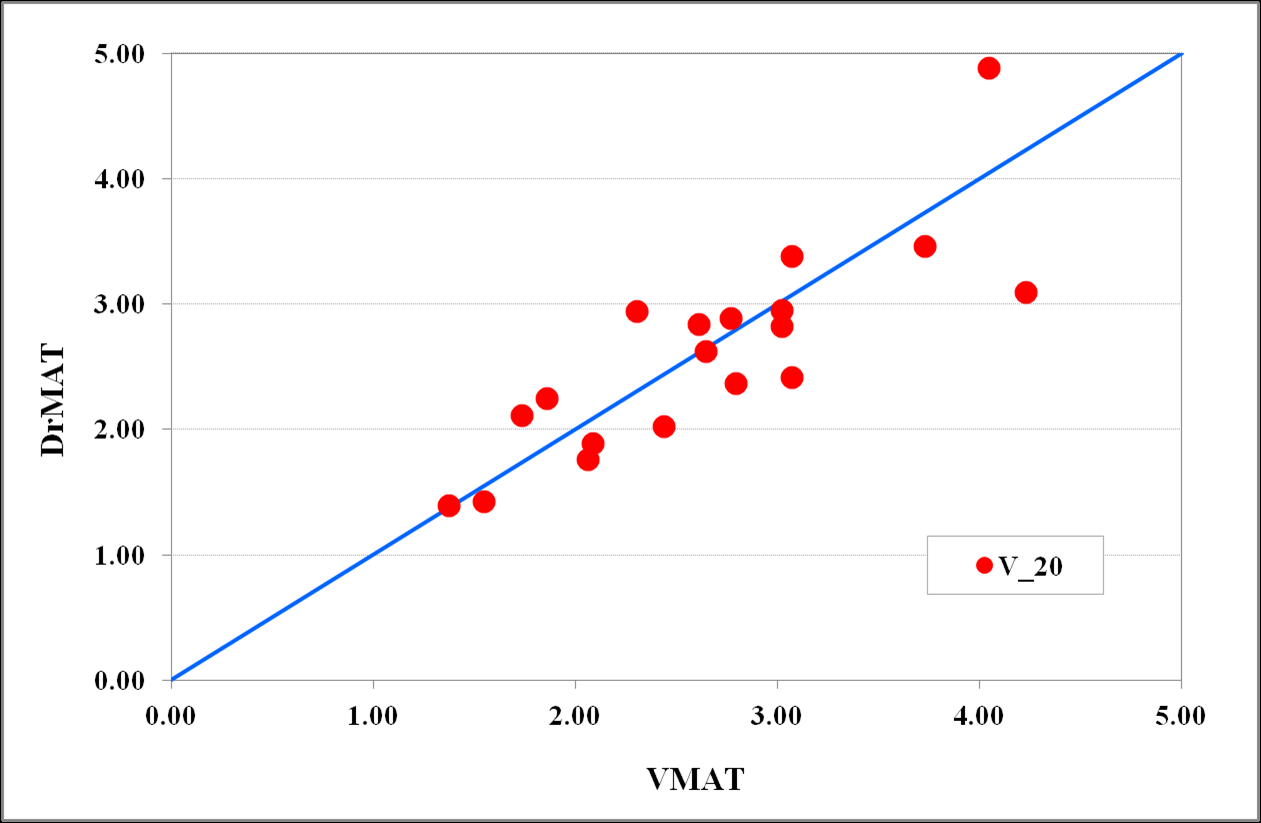

Supplement: S5 Fig — (TIF) [file pone.0158053.s005.tif]
